# Concentration–QTc analysis of quizartinib in patients with relapsed/refractory acute myeloid leukemia

**DOI:** 10.1007/s00280-020-04204-y

**Published:** 2021-01-08

**Authors:** Dongwoo Kang, Elizabeth Ludwig, David Jaworowicz, Hannah Huang, Jill Fiedler-Kelly, Jorge Cortes, Siddhartha Ganguly, Samer Khaled, Alwin Krämer, Mark Levis, Giovanni Martinelli, Alexander Perl, Nigel Russell, Malaz Abutarif, Youngsook Choi, Ophelia Yin

**Affiliations:** 1 Daiichi Sankyo, Inc, Basking Ridge, NJ USA; 2 Cognigen Corporation, a Simulations Plus Company, Buffalo, NY USA; 3 Georgia Cancer Center at Augusta University, Augusta, GA USA; 4 University of Kansas Cancer Center, Fairway, KS USA; 5 City of Hope, Arcadia, CA USA; 6 Clinical Cooperation Unit Molecular Hematology/Oncology, Department of Internal Medicine V, University of Heidelberg, Heidelberg, Germany; 7 The Sidney Kimmel Comprehensive Cancer Center at Johns Hopkins University, Baltimore, MD USA; 8 Istituto Scientifico Romagnolo per lo Studio e la Cura dei Tumori (IRST) IRCCS, Meldola, Italy; 9 Division of Hematology and Oncology, Abramson Cancer Center, University of Pennsylvania, Philadelphia, PA USA; 10 Centre for Clinical Haematology, Nottingham University Hospital, Nottingham, UK

**Keywords:** Concentration–QTc analysis, Quizartinib, AC886, Relapsed/refractory, Acute myeloid leukemia

## Abstract

**Purpose:**

This analysis evaluated the relationship between concentrations of quizartinib and its active metabolite AC886 and QT interval corrected using Fridericia’s formula (QTcF) in patients with relapsed/refractory acute myeloid leukemia (AML) treated in the phase 3 QuANTUM-R study (NCT02039726).

**Methods:**

The analysis dataset included 226 patients with AML. Quizartinib dihydrochloride was administered as daily doses of 20, 30, and 60 mg. Nonlinear mixed-effects modeling was performed using observed quizartinib and AC886 concentrations and time-matched mean electrocardiogram measurements.

**Results:**

Observed QTcF increased with quizartinib and AC886 concentrations; the relationship was best described by a nonlinear maximum effect (*E*_max_) model. The predicted mean increase in QTcF at the maximum concentration of quizartinib and AC886 associated with 60 mg/day was 21.1 ms (90% CI, 18.3–23.6 ms). Age, body weight, sex, race, baseline QTcF, QT-prolonging drug use, hypomagnesemia, and hypocalcemia were not significant predictors of QTcF. Hypokalemia (serum potassium < 3.5 mmol/L) was a statistically significant covariate affecting baseline QTcF, but no differences in ∆QTcF (change in QTcF from baseline) were predicted between patients with versus without hypokalemia at the same quizartinib concentration. The use of concomitant QT-prolonging drugs did not increase QTcF further.

**Conclusion:**

QTcF increase was dependent on quizartinib and AC886 concentrations, but patient factors, including sex and age, did not affect the concentration–QTcF relationship. Because concomitant strong cytochrome P450 3A (CYP3A) inhibitor use significantly increases quizartinib concentration, these results support the clinical recommendation of quizartinib dose reduction in patients concurrently receiving a strong CYP3A inhibitor.

**Clinical Trial Registration:**

NCT02039726 (registered January 20, 2014).

**Supplementary Information:**

The online version contains supplementary material available at 10.1007/s00280-020-04204-y.

## Introduction

FMS-like tyrosine kinase 3 (FLT3) has emerged as a rational therapeutic target in acute myeloid leukemia (AML). FLT3 is normally expressed in hematopoietic progenitor cells, and signaling through FLT3 promotes proliferation and differentiation [1]. A *FLT3* mutation occurs in approximately 30% of patients with AML [1, 2]. The *FLT3* internal tandem duplication (ITD) mutation represents the most common type of *FLT3* mutation and is associated with high relapse rates, decreased response to salvage therapy, and shorter overall survival when compared with *FLT3* wild-type disease [1–4].

Quizartinib dihydrochloride is an oral, once-daily, highly potent and selective, type II tyrosine kinase inhibitor targeting FLT3 that has shown clinical activity in patients with *FLT3*-ITD-positive relapsed/refractory AML [5, 6]. Quizartinib is primarily metabolized by cytochrome P450 3A (CYP3A), and its major metabolite, AC886, is biologically active against FLT3 and is also a substrate of CYP3A [7]. The peak plasma concentrations of quizartinib and AC886 occur at approximately 4 and 5 h, respectively. The exposure is dose proportional from 20 to 90 mg. At steady state, AC886 exposure is approximately 60% of that of quizartinib. Quizartinib and AC886 have estimated effective half-lives of 73 and 119 h, respectively.

Quizartinib at 1680 ng/mL (3 μM) and AC886 at 1730 ng/mL (3 μM) inhibited the human ether-a-go-go-related gene (hERG) current by 16.4% and 12.0%, respectively. Quizartinib, at concentrations ranging from 56.1 ng/mL (0.1 μM) to 1630 ng/mL (2.9 μM), inhibited the slowly activating component of delayed rectifier potassium currents (IKs), with a maximum inhibition of 67.5% (at 1630 ng/mL [2.9 μM]). Although quizartinib and AC886 inhibited both hERG current and IKs, the predominant effect was on IKs.

Dose-dependent QT prolongation is a major adverse event associated with quizartinib. The phase 1 dose-escalation study initially identified 200 mg/day as the maximum tolerated dose of quizartinib, with QT prolongation being the dose-limiting toxicity [8]. In a subsequent phase 2 study, quizartinib 200 mg daily was the initial dose regimen; however, 12 of 17 patients who received this dose exhibited a QT interval corrected using Fridericia’s formula (QTcF) of > 480 ms, and 14 patients had a change in QTcF from baseline (ΔQTcF) of 60 ms above baseline. Therefore, the dose was subsequently reduced to 135 or 90 mg/day for all patients [5].

Another phase 2 study, which evaluated 2 dosing regimens of quizartinib, found that either 30 or 60 mg/day (with escalations to 60 or 90 mg/day, respectively, permitted for lack or loss of efficacy) demonstrated clinical activity, with a lower rate of QT prolongation observed than in the prior studies evaluating higher doses. QT prolongation remained dose dependent and was substantially reduced at lower doses [6]. In a drug–drug interaction study, co-administration of ketoconazole, a strong CYP3A inhibitor, at 200 mg twice daily resulted in an increase in quizartinib steady-state area under the concentration–time curve (AUC) and maximum concentration (*C*_max_) of 96% and 86%, respectively, and a decrease in AC886 steady-state AUC and *C*_max_ of 14% and 18%, respectively. Co-administration of fluconazole, a moderate CYP3A inhibitor, at 200 mg twice daily caused a minor change in these exposures [9].

In the phase 3 QuANTUM-R study (NCT02039726), patients with relapsed/refractory *FLT3*-ITD–positive AML were randomized to standard chemotherapy or single-agent quizartinib administered at 60 mg/day with a 30-mg/day lead-in (or 30 mg/day with a 20-mg/day lead-in for patients taking a strong CYP3A inhibitor). This study demonstrated a statistically significant overall survival benefit with quizartinib in patients with relapsed/refractory *FLT3*-ITD–positive AML versus salvage chemotherapy, with a 24% reduction in the risk of death during the observation period. Grade 3 QT prolongation was uncommon, and there were no occurrences of torsades de pointes or other grade 4 QT-prolongation events [10]. On the basis of the results from the QuANTUM-R study and a phase 2 study in Japan in patients with relapsed/refractory *FLT3*-ITD–positive AML, quizartinib was approved in Japan in June 2019 [11].

The objectives of this analysis were to (1) characterize the relationship between concentrations of quizartinib and its active metabolite AC886 and QTcF and (2) identify significant covariates that affect the exposure–response relationship using the observed data in the QuANTUM-R study.

## Materials and methods

### Dataset

The concentration–QTc (C-QTc) analysis included data from the phase 3 QuANTUM-R study, which was conducted at 152 sites in 19 countries [10]. The institutional review board or ethics committee at each site approved the study protocol, and all patients provided written informed consent per the Declaration of Helsinki and Good Clinical Practice.

In QuANTUM-R, the starting dose of quizartinib was 30 mg/day (26.5 mg free base), followed by an increase to 60 mg/day (53.0 mg free base) after 2 weeks if QTcF was ≤ 450 ms. Patients receiving a concurrent strong CYP3A inhibitor initiated quizartinib at 20 mg/day (17.7 mg free base), with an increase to 30 mg/day (26.5 mg free base) after 2 weeks if QTcF was ≤ 450 ms. The dose was reduced from 60 to 30 mg daily or from 30 to 20 mg daily if protocol-specified criteria for dose reduction were met (for example, QTcF > 480 ms, persistent grade ≥ 3 nonhematologic toxicity, or myelosuppression in patients achieving complete remission with incomplete platelet recovery or complete remission with incomplete hematologic recovery who had received ≥ 2 cycles of treatment).

Plasma concentrations of quizartinib and AC886 were measured by BASi (West Lafayette, IN, USA) using a validated liquid chromatography–tandem mass spectrometry method. The analytical range validated was from 2 to 2000 ng/mL for both quizartinib and AC886. For quizartinib, the ranges for within-run and between-run assay precision were 0.9 to 6.3% and 3.0 to 6.3%, respectively, for quality control samples at concentrations within the calibration curve; the accuracy ranged from − 11.2 to 7.7% and − 4.0 to 4.0%, respectively. The lower limit of quantification was 2 ng/mL. For AC886, the ranges for within-run and between-run assay precision were 0.3 to 7.4% and 2.2 to 6.7%, respectively, for quality control samples at concentrations within the calibration curve; the accuracy ranged from − 9.0 to 6.7% and − 3.0 to 2.1%, respectively. The lower limit of quantification was 2 ng/mL.

For each patient, centrally read triplicate QTc measurements (from three 12-lead ECGs, ≥ 5 min apart per time point) were obtained at screening; predose and 2, 4, and 6 h postdose on days 1 and 15 of cycle 1; predose and 2 to 4 h postdose on days 2 and 8 of cycle 1 and on day 1 of cycles 2 and 3; and at any time on day 1 of subsequent cycles. Pharmacokinetic (PK) blood samples were collected at each of the QTc measurement times after ECG was performed, until cycle 3 of treatment.

For the C–QTc analysis, QTc data and concentration records were matched by comparing the actual date/time of each replicate QTc measurement versus the date/time of the corresponding concentration measurement. The difference in time between the 2 measurements was computed and used to determine whether the replicate QTc measurements met the criteria to be matched with concentration. A difference of ≤ 30 min was allowed for all time points except for the nominal 24-h postdose QTc measurements, in which up to 90 min was allowed.

The demographic and clinical covariates evaluated for their effect on the C–QTc model parameters included age, baseline body weight, sex, race, baseline QTc, coadministration of QT-prolonging drugs, and selected electrolyte deficiencies (hypomagnesemia [serum magnesium concentration < 0.75 mmol/L], hypokalemia [serum potassium concentration < 3.5 mmol/L], and hypocalcemia [serum calcium concentration < 2.2 mmol/L]). For patients with serum albumin concentrations < 4.0 g/dL, the total serum calcium concentration was corrected according to the standard formula [12].

All exploratory data analyses were performed using SAS 9.4 (SAS Institute Inc) and KIWI Version 2 (Cognigen Corporation). The C–QTc modeling was performed using the computer program NONMEM Version 7.3.0 (ICON Development Solutions).

### C–QTc analysis

Exploratory data analyses and data visualization were used to determine the characteristics of the data, assess possible trends, confirm the appropriateness of the models tested, and verify model assumptions. These included inspection of QTc measurements versus time, concentration, and *R*–*R* interval (duration of ECG interval between consecutive waves) as well as the possible time delay between QTc and concentration (i.e., hysteresis).

The first-order conditional estimation method, with interaction as implemented in NONMEM, was used for the C–QTc analysis. QTcF data were modeled using quizartinib and AC886 concentrations as independent variables. The base model included the baseline term to estimate the typical value of baseline QTcF along with interindividual variability. To account for circadian variation, the time effect on baseline QTcF was also included in the model. Briefly, baseline QTcF data were split into 10 bins according to 24-h clock time (each bin had approximately the same number of samples), and the mean differences in QTcF for the 10 bins of time were estimated by the model. For determination of the structural model, various functions of quizartinib and AC886 concentrations were evaluated, with interindividual variability estimated for applicable parameters. Explored models included a linear model having separate slopes for quizartinib and AC886 concentrations and a maximum effect (*E*_max_) model of quizartinib and AC886 concentrations, with and without a Hill coefficient, to account for sigmoidicity. Once the C–QTc model for QTcF was determined, ∆QTcF was obtained by subtracting the baseline terms.

Model selection was determined using several criteria, including decrease in NONMEM objective function value, inspection of goodness-of-fit plots, and decrease in estimates of interindividual variability (IIV) and/or residual variability (RV). Prediction-corrected visual predictive checks (pcVPCs) were performed for major steps during the model-building process. A detailed description of the covariate modeling and model building process is available in Online Resource 1.

The appropriateness of IIV and RV models was reevaluated during model refinement after covariate analysis. This procedure included reassessment of the assumed distributional form (normal or log-normal) of each IIV term as well as the functional form (additive or proportional) of the RV model. A number of models were evaluated, and the best model was selected based on numerical and graphical criteria.

The final C–QTc model was utilized to obtain the model-predicted mean ΔQTcF and 90% confidence interval (CI) at the maximum concentration at steady state (*C*_max,ss_). Individual *C*_max,ss_ values for patients enrolled in QuANTUM-R who received quizartinib 60 mg were generated using the population PK model [13], then the geometric mean of quizartinib *C*_max,ss_ was obtained. When making predictions of ΔQTcF, the bootstrap method was used to account for multiple parameters in the model and the correlation between the parameters [14]. First, 1000 random samples of C–QTc model parameters were generated from the mean and covariance matrix of the final C–QTc model. Then, corresponding ΔQTcF values were computed at the *C*_max,ss_; 90% CI was obtained by the 5th and 95th percentiles from the distribution.

## Results

### Data

After matching QTcF data and concentration records using the actual date/time, a total of 2842 time-matched mean QTcF and quizartinib and AC886 concentration measurement records from 226 patients were available for the C–QTc analysis. The overall safety analysis dataset for QuANTUM-R included 241 patients; 15 were excluded from the C–QTc dataset. The reasons for exclusion were no matching concentration, no postbaseline QTcF observation, no baseline QTcF observation, and incorrect dose record. These reasons for exclusion were atypical; hence, these exclusions would not systematically bias the analysis results. Actual PK/QTc sampling dates and times were converted from clock times to decimal times for use in the analysis (e.g., midnight = 0, 8:30 am = 8.5). The average time difference between the mean QTcF and concentration measurement records was − 5.28 min, as calculated by ECG time minus PK time.

The majority of patients were white (74.3%), with slightly more women (54.0%) than men. Patient age ranged from 19 to 81 years, with a median of 55 years, and body weight ranged from 39.5 to 147 kg, with a median of 70.0 kg. The median value for baseline QTcF in the analysis population was 414 ms (Table 1).

**Table 1:** Patient characteristics in the C–QTc dataset

Characteristic	*N* = 226
Age, median (range), years	55 (19, 81)
Sex, *n* (%)	
Male	104 (46.0)
Female	122 (54.0)
Race, *n* (%)	
White	168 (74.3)
Black or African American	8 (3.5)
Asian	24 (10.6)
American Indian or Alaskan Native	1 (0.4)
Other	7 (3.1)
Unknown	18 (8.0)
Weight, median (range), kg	70.0 (39.5, 147)
Baseline QTcF, median (range), ms	414 (364, 471)
Hypocalcemia, *n* (%)	97 (42.9)
Hypokalemia, *n* (%)	35 (15.5)
Hypomagnesemia, *n* (%)	63 (27.9)
QT-prolonging drug use, *n* (%)	66 (29.2)

*C–QTc* concentration–QTc; *QTcF* QT interval corrected using Fridericia’s formula, *SD* standard deviation

**Fig. 1:**
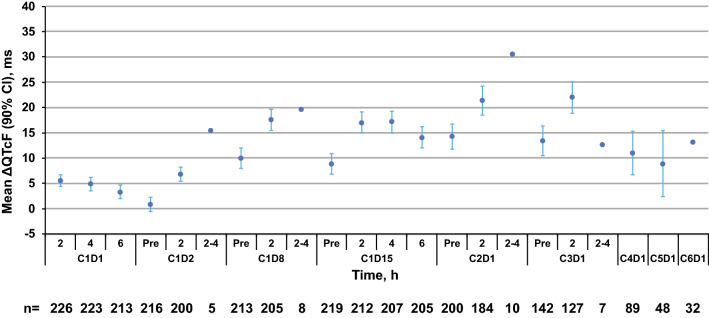
Observed QTcF data. The mean and 90% confidence interval are shown per visit: *C* cycle, *D* day

Figure 1 shows the observed mean ΔQTcF per visit from the QuANTUM-R study. All tested covariates were baseline values except for the use of QT-prolonging drugs and serum electrolyte imbalances, such as hypocalcemia, hypokalemia, and hypomagnesemia, which were tested as time-varying covariates.

### Exploratory analysis

QTcF was chosen as the preferred dependent variable for this analysis because it was used for dose increase/reduction criteria in the clinical study. Furthermore, the Fridericia correction method was recommended in a recent white paper on QTc analysis [14]. To confirm that the QT interval was properly corrected, scatterplots of triplicate QTcF values from pre- and post-treatment versus *R*–*R* interval, stratified by sex, were examined (Online Resource 2). The results suggested that QTcF was nearly independent of *R*–*R* interval and confirmed the use of QTcF as an adequate correction of QT interval in this population. Notably, QTcF did not increase when heart rate increased. Approximately 10% of patients had a > 25% increase in heart rate from baseline as well as a heart rate ≥ 100 beats per minute (i.e., *R*–*R* interval ≤ 600 ms) measured from centrally read ECGs, demonstrating an adequate number of patients to assess the effect of elevated heart rates on QTcF.

No consistent trend for hysteresis was observed in the drug concentration versus QTcF plot. Therefore, a direct relationship between drug concentration and QTcF was considered appropriate and was used as the base model structure.

### Model building

To investigate possible variations in the QTc data, pretreatment QTcF data were plotted according to 24-h clock time using 10 bins with approximately the same number of samples in each bin. The plot showed considerable variation in pretreatment QTcF values versus time before administration of quizartinib, which was attributed to circadian rhythm. Therefore, the use of baseline correction was necessary. Because the majority of pretreatment QTc measurements in QuANTUM-R were obtained between 8:00 am and 5:00 pm, there were insufficient pretreatment data to support the inclusion of a full 24-h circadian rhythm function for the correction of baseline. Instead, a fixed time effect model for baseline correction was implemented [14, 15] by estimating the mean QTcF difference for each of the 10 bins (Online Resource 3).

**Fig. 2:**
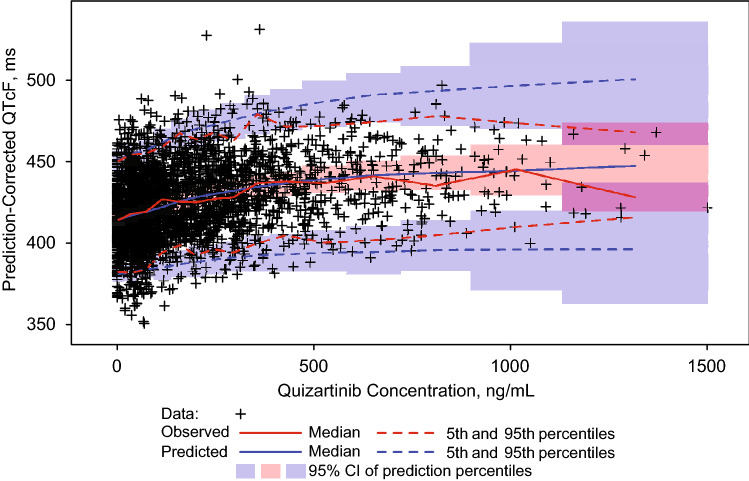
Prediction-corrected visual predictive checks for the final QTcF model. *CI* confidence interval, *QTcF* QT interval corrected using Fridericia’s formula

To determine the structural model, a linear model with different slopes for quizartinib and AC886 and a sigmoid *E*_max_ model with separate *E*_max_ terms for quizartinib and AC886 were evaluated. The sigmoid *E*_max_ model had a lower Akaike information criterion (AIC) value than the linear model (AIC: 17,628.623 vs 17,784.369), supporting a better fit to the data than the linear model. This corroborated the trend exhibited in the exploratory data analysis plots, in which a possible plateauing of QTcF increase was observed at the higher concentration range. Therefore, the C–QTc data were modeled with *E*_max_ functions of quizartinib and AC886, including a baseline term and interindividual variabilities on *E*_max_ and baseline terms. Covariate analysis identified hypokalemia as a statistically significant predictor of baseline QTcF in the C–QTc model, with both quizartinib and AC886 concentrations as predictors of response. Further evaluation of IIV and RV functional forms during model refinement suggested that the model with log-normal distribution of IIV for the intercept, normal distribution of IIV for the *E*_max_ terms, and proportional RV best fit the data, as indicated by a lower objective function value and better precision of parameter estimates (Online Resource 4). The final model parameter estimates, along with corresponding precisions (relative standard error expressed as a percentage [% RSE]) for the final C–QTc model, are presented in Table 2. The model parameters for baseline QTcF, *E*_max_ for quizartinib, half maximal effective concentration (EC_50_) for both quizartinib and AC886, and gamma for quizartinib were estimated with good precision (< 20% RSE), while the *E*_max_ and gamma for AC886 and the effect of hypokalemia on baseline QTcF were estimated with moderate precision (44.5%, 54.3%, and 31.5% RSE, respectively). Random effect parameters, IIV and RV, were estimated with good precision (≤ 30% RSE). The goodness-of-fit plots were reasonably unbiased and suggested that the model was able to characterize the observed C–QTc relationship well (Daiichi Sankyo, Inc. Data on file). The pcVPCs were performed by generating 1000 replicates of the analysis dataset. The pcVPC plot shown for quizartinib concentration, which plays a major role in QTc prolongation, indicates good concordance between observed data and model predictions (Fig. 2).

**Table 2:** Parameter estimates of final C–QTc model effects of both quizartinib and AC886 concentrations

Parameter	Final parameter estimate		Interindividual variability	
	Typical value	% RSE	Magnitude	% RSE
Baseline QTcF, ms	413	0.325	4.42% CV	9.81
Fractional change in baseline for hypokalemia	0.0149	31.5		
Fixed time effect for 1st decile of clock time	1.46	69.2		
Fixed time effect for 2nd decile of clock time	− 0.853	117		
Fixed time effect for 3rd decile of clock time	0	Fixed		
Fixed time effect for 4th decile of clock time	1.89	55.0		
Fixed time effect for 5th decile of clock time	3.06	31.0		
Fixed time effect for 6th decile of clock time	4.12	21.1		
Fixed time effect for 7th decile of clock time	5.06	22.1		
Fixed time effect for 8th decile of clock time	3.56	29.4		
Fixed time effect for 9th decile of clock time	3.20	32.0		
Fixed time effect for 10th decile of clock time	1.56	72.8		
Maximum effect of parent drug, ms	31.2	14.5	21.6 (SD)	29.7
Concentration at 50% of maximum parent effect, ng/mL	315	19.4	NE	NA
Hill coefficient for parent drug	1.52	10.9	NE	NA
Maximum effect of drug metabolite, ms	2.69	44.5	12.3 (SD)	15.6
Concentration at 50% of maximum metabolite effect, ng/mL	60.5	9.14	NE	NA
Hill coefficient for drug metabolite	16.9	54.3	NE	NA
Residual variability	2.48% CV	6.58		
Minimum value of the objective function = 17,542.877				

*AC886* compound code for active metabolite of quizartinib, *C–QTc* concentration–QTc, *ms* milliseconds, *%CV* coefficient of variation expressed as a percentage, *NA* not applicable, *NE* not estimated, *QTcF* QT interval corrected using Fridericia’s formula, *% RSE* relative standard error expressed as a percentage, *SD* standard deviation

The equation for QTcF based on the final model is shown below:

$$\begin{gathered} {\text{QTcF}}_{{it}} = \left( \begin{gathered} \left( {413 \times (1 + 0.0149 \times {\text{flag}}_{{{\text{hypok}}_{{i,t}} }} + p_{t} } \right) \times e^{{\eta _{{i,{\text{base}}}} }} \hfill \\ + \frac{{\left( {31.2 + \eta _{{i,E{\text{max}},p}} } \right) \times {\text{Cp}}(t)_{{i,p}} ^{{1.52}} }}{{315^{{1.52}} + {\text{Cp}}(t)_{{i,p}} ^{{1.52}} }} + \frac{{\left( {2.69 + \eta _{{i,E{\text{max}},m}} } \right) \times Cp(t)_{{i,m}} ^{{16.9}} }}{{60.5^{{16.9}} + {\text{Cp}}(t)_{{i,m}} ^{{16.9}} }} \hfill \\ \end{gathered} \right) \hfill \\ \quad \quad \quad \times e^{{\varepsilon _{{it}} }} \hfill \\ \end{gathered}$$

where QTcF_*it*_ is the observed QTcF value in the *i*th patient at time *t*; *p*_*t*_ is the fixed time effect on QTcF; flag_hypok*i,t*_ is the indicator variable for hypokalemia in the *i*th patient at time *t* (1 = with hypokalemia, 0 = without hypokalemia); $\eta_{i}$ is the between-patient random effect parameter assumed to be normally distributed with mean zero and variance Ω; ${\text{Cp}}(t)_{i,p}$ is the plasma concentration of quizartinib (ng/mL) at time *t* in the *i*th patient; ${\text{Cp}}(t)_{i,m}$ is the plasma concentration of AC886 (ng/mL) at time *t* in the *i*th patient; and $\varepsilon_{it}$ is the residual within-patient variability assumed to be normally distributed with mean zero and variance σ^2^. ${p}_{t}$ is modeled as:

$${p}_{t}=\theta \times I\left[\mathrm{time}=t,t\ne 0\right]$$

where:

$\theta$ is the fixed time effect shift on the intercept for each bin; and ${\rm I}[\mathrm{time}=t,t\ne 0]$ is the indicator variable, with the value of 1 if the condition in brackets is true and 0 if otherwise.

### Model fitting

**Fig. 3:**
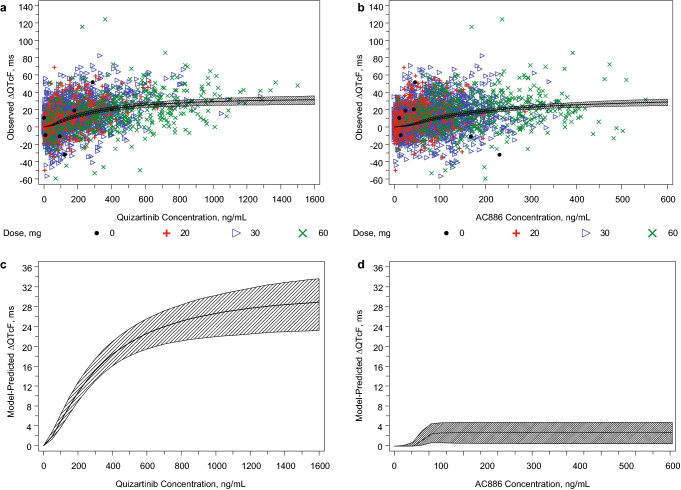
Top: ∆QTcF versus concentration for **a** quizartinib and **b** AC886. The solid line represents the model-predicted median drug effect predictions; the shaded area represents the 90% uncertainty around median drug effect predictions. Predicted ∆QTcF represents contributions of quizartinib and AC886. Bottom: Contribution of quizartinib and AC886 to ∆QTcF. The solid line represents the median model-predicted ∆QTcF with (**c**) quizartinib only and (**d**) AC886 only; the shaded area represents the 90% uncertainty around median drug effect predictions. *QTcF* QT interval corrected using Fridericia’s formula

Figure 3 shows the model-predicted ΔQTcF and 90% CI overlaid with the observed ΔQTcF across the range of quizartinib (Fig. 3a) and AC886 (Fig. 3b) concentrations. The model-predicted ΔQTcF includes effects from both quizartinib and AC886. Additionally, Fig. 3 shows the model-predicted ΔQTcF effect of quizartinib alone (Fig. 3c) and AC886 alone (Fig. 3d), with corresponding 90% CI, demonstrating the relative contribution of quizartinib versus that of AC886 in QT prolongation.

The geometric mean *C*_max,ss_ of quizartinib was 401 ng/mL, and the corresponding geometric mean AC886 concentration at the time of the quizartinib *C*_max,ss_ was 204 ng/mL in patients in QuANTUM-R who received quizartinib 60 mg daily. The model-predicted mean ΔQTcF at these geometric mean *C*_max,ss_ values for quizartinib 60 mg daily was 21.1 ms (90% CI, 18.3–23.6 ms). Consequently, quizartinib belongs to the class of drugs that have increased QT-prolongation risk according to the US Food and Drug Administration E14 guidance [16]. Histograms of ∆QTcF at the geometric mean *C*_max,ss_ values of quizartinib and AC886 are provided in Online Resource 5. At the geometric mean *C*_max,ss_ of quizartinib, 51.5% and 77.6% of the observations were above 20 and 10 ms, respectively. At the geometric mean *C*_max,ss_ of AC886, 41.2% and 67.2% of the observations were above 20 and 10 ms, respectively.

Approximately 73% of patients (*n* = 177) treated with quizartinib in QuANTUM-R used QT-prolonging drugs as deemed necessary by their treating physicians. A list of these agents together with their categories is provided in Online Resource 6. However, a significant effect of concomitant QT-prolonging drug use on QTcF was not identified in the C–QTc analysis. The lack of a significant effect warranted additional confirmation. An analysis of duration of overlap in centrally reviewed QTcF data for patients who received ≥ 1 dose of QT-prolonging drugs concomitantly with quizartinib was performed (mean duration of overlap, 96 days; maximum duration of overlap, 934 days). This analysis showed that neither the magnitude of QTcF elevation nor the frequency of an increase of > 60 ms from baseline (range, 3.4–4.0%) was changed by the duration of overlap. Further assessment was conducted using data from 60 patients having matched concentrations and ECG measurements during the time of concomitant administration of QT-prolonging drugs and during the time when these same patients were not taking QT-prolonging drugs. This allowed for a within-patient evaluation of the C–QTc relationship in the presence and absence of QT-prolonging drugs. The results of this analysis suggested that concomitant QT-prolonging drugs did not further increase QTcF during the period of their administration (Online Resource 7). However, limitations exist with the analysis dataset, where the dose and duration of administration of those QT-prolonging drugs were not taken into consideration.

## Discussion

The relationship between time-matched concentrations of both quizartinib and its major metabolite AC886 and QTcF measurements was described using a model composed of 2 sigmoid *E*_max_ expressions (separately describing the effect of quizartinib and AC886 exposures) that was developed with data obtained from multiple-dose administration of quizartinib (20, 30, and 60 mg once daily) in patients with AML in the QuANTUM-R study [10]. The C–QTc model was parameterized in terms of baseline QTcF; fixed time effect parameters for the correction of baseline QTcF; and separate *E*_max_, EC_50_, and Hill coefficient values for quizartinib and AC886. Both parent quizartinib and metabolite AC886 concentrations were included in the model, in accordance with the recommendations given in a recent white paper on C–QTc analysis [14, 17].

An oscillatory function extending over a 24-h period has been used for circadian rhythm correction in C–QTc analysis [18], but it requires frequent ECG sampling over the full 24-h time frame for appropriate application. For this C–QTc analysis, a fixed time effect approach was adopted because pretreatment QTcF data were obtained primarily between 8:00 am and 5:00 pm. A fixed time effect model corrects the baseline for circadian rhythm for the time span for which observed data are available, rather than generating a 24-h circadian rhythm that extends over the period when there may be limited or no observed data.

Quizartinib inhibits IKs with an IC_50_ < 300 nM (Daiichi Sankyo, Inc. Data on file). Owing to the concern that QT interval would fail to shorten during sudden tachycardia if IKs is impaired, QTcF was investigated versus *R*–*R* interval. However, there was no evidence of QTcF prolongation with increased heart rate in both men and women. This relationship between QTcF and *R*–*R* interval appeared to be the same before and during treatment with quizartinib (Online Resource 2). The relationship was also observed with uncorrected QT.

The effects of patient demographics, electrolyte concentrations, and selected concomitant medication use on QTcF variability were evaluated using the C–QTc model. These covariate effects were tested on baseline QTcF, quizartinib *E*_max_, and AC886 *E*_max_. Covariate modeling was not performed on the EC_50_ of quizartinib or AC886 because IIV in EC_50_ was not estimated. In the final model, hypokalemia was identified as a significant covariate affecting baseline QTcF but was not found to be a predictor of *E*_max_ parameters. In patients with hypokalemia, the mean QTcF at baseline was predicted to be prolonged by 6.15 ms relative to the typical baseline value of 413 ms in patients without hypokalemia. Since the effect of hypokalemia was observed on baseline QTcF and not on *E*_max_, it is expected that the ΔQTcF would be the same at the same quizartinib and AC886 concentrations in patients with or without hypokalemia.

Abnormal serum potassium levels, both hypokalemia and hyperkalemia, affect cardiac function [19]. In the C–QTc dataset, no patients had hyperkalemia (defined as serum potassium concentration > 5.3 mmol/L) at baseline, and only 2.7% of patients experienced hyperkalemia during the study. In contrast, hypokalemia (defined as serum potassium concentration < 3.5 mmol/L) was experienced by 15.5% of patients at baseline and 33.6% during the study. When tested as a continuous covariate, serum potassium concentration was not significant for the C–QTc model. The dichotomous hypokalemia variable was found to be significant and more informative than continuous serum potassium concentration values and therefore was used in the C–QTc model for quantifying the effect of a low level of serum potassium.

The final model estimates of *E*_max_ were 31.2 and 2.69 ms for quizartinib and AC886, respectively. The relative standard errors associated with the *E*_max_ estimates were reasonable for quizartinib (at 14.5% coefficient of variation [CV]) but high for AC886 (at 44.5% CV). The ratio of quizartinib *E*_max_ to AC886 *E*_max_ was approximately 12:1, showing a much greater effect of quizartinib on QTcF relative to AC886.

Previously, a C–QTc analysis was performed in 73 patients with relapsed/refractory AML receiving quizartinib in the phase 2 study 2689-CL-2004 [6, 20]. In this prior analysis, a linear C–QTc model for quizartinib and AC886 was selected as the final model. By comparison, there was a tendency toward reaching a plateau in the C–QTc relationship in the QuANTUM-R QTcF data, which may have been due to the QT-based dose-adjustment scheme (aimed at mitigating the potential for QT prolongation) implemented in the trial. When different structural models were explored using the QuANTUM-R dataset, the sigmoid *E*_max_ model was selected as the final model, as it fit the QTcF data better and was more statistically significant than a linear model. The superiority of the sigmoid *E*_max_ model over the linear model lies in its ability to more accurately characterize the C–QTc relationship at the higher end of the concentration range. When a sensitivity analysis was performed and predictions were compared, the linear model showed predictions of ΔQTcF similar to those of the sigmoid *E*_max_ model for the quizartinib and AC886 concentration ranges of < 700 ng/mL and < 400 ng/mL, respectively (Online Resource 8). Although the selection of a linear model to describe data from the 2689-CL-2004 study and the selection of a sigmoid *E*_max_ model to describe data from the QuANTUM-R study were guided by statistical criterion values unique to each analysis dataset, both models produced adequate and similar predictions of ΔQTcF for the clinically relevant concentration ranges of quizartinib and AC886.

QT prolongation and arrythmia risks are generally expected to increase when QT-prolonging drugs are used, although this assumption is challenging to rigorously confirm in studies given the rare nature of torsades de pointes, even in the setting of QT prolongation. Meid et al. concluded that combinations of QT-prolonging drugs did not necessarily result in additive QT prolongation in their evaluation of 2558 psychiatric inpatients and outpatients using the Arizona Center for Education and Research on Therapeutics class of co-prescribed QT-prolonging drugs [21].

In QuANTUM-R, the coadministration of QT-prolonging drugs with quizartinib did not have a detectable impact on QT interval prolongation. Further analyses in the 177 patients using QT-prolonging drugs concomitantly in QuANTUM-R showed that magnitude of QTcF elevation—and frequency of an increase of > 60 ms from baseline—was not changed by the duration of overlap with quizartinib treatment. In addition, within-patient evaluation of the C–QTc relationship in the presence and absence of QTc-prolonging drugs suggested that concomitant administration of these drugs did not result in a further increase in QTcF during the period of their administration (Online Resources 6 and 7). However, the analysis dataset had limitations due to the lack of information on dose and duration of administration of the QT-prolonging drugs.

Collectively, these findings demonstrate that the use of concomitant QT-prolonging medication does not affect the relationship between QTcF and quizartinib concentration. Possible reasons for such a lack of additive QT-prolonging effect could include diverse mechanisms leading to QT prolongation, differing drug effects on the various cardiac ion channels involved, and a differing magnitude of QT effect relative to drug dose or variability in the QT interval. Regardless of the reason, these findings suggest that patients who receive quizartinib according to the risk mitigation strategy used in QuANTUM-R (i.e., exclusion of patients at high risk for QT prolongation, utilization of a QT-based dosing regimen, use of ECG monitoring, and maintenance of normal electrolyte levels) can receive concomitant QT-prolonging drugs without increasing the risk of QT prolongation.

The increase in quizartinib exposure with concomitant use of strong CYP3A inhibitors was demonstrated in a drug–drug interaction study and in a population PK analysis. In the drug–drug interaction study, the quizartinib steady-state AUC and *C*_max_ were predicted to increase by 96% and 86%, respectively, with concomitant administration of the strong CYP3A inhibitor ketoconazole [9]. In the population PK analysis, quizartinib AUC increased by 82% and *C*_max_ increased by 72% with concomitant use of a strong CYP3A inhibitor [13]. Because the current analysis demonstrated a positive exposure–response relationship in C–QTc, these results suggest that dose reduction is necessary for patients who are receiving a concomitant strong CYP3A inhibitor to minimize the risk of QT prolongation. This method was demonstrated to be effective in QuANTUM-R, where grade 3 QT prolongation was infrequent and there were no instances of grade 4 events in the quizartinib arm [10]. As patients’ intrinsic factors, such as sex and age, do not appear to affect the exposure–response C–QTc relationship, the only factor that was shown to result in QTcF increase was an increase in quizartinib exposure.

In conclusion, an exposure–response relationship was demonstrated in this C–QTc analysis of quizartinib using an *E*_max_ function. With quizartinib exposure as a significant predictor of QT increase, this analysis supports the clinical recommendation that dose reduction is necessary to reduce the risk of QT prolongation in patients receiving concomitant strong CYP3A inhibitors and in patients experiencing increased QT interval (QTcF > 450 ms).

## Supplementary Information

Below is the link to the electronic supplementary material.

Supplementary file 1 (PDF 559 KB)

## References

[1] Daver N, Schlenk RF, Russell NH, Levis MJ (2019). Targeting FLT3 mutations in AML: review of current knowledge and evidence. Leukemia.

[2] Levis M (2013). FLT3 mutations in acute myeloid leukemia: what is the best approach in 2013?. Hematol Am Soc Hematol Educ Prog.

[3] Boissel N, Cayuela JM, Preudhomme C, Thomas X, Grardel N, Fund X, Tigaud I, Raffoux E, Rousselot P, Sigaux F, Degos L, Castaigne S, Fenaux P, Dombret H (2002). Prognostic significance of FLT3 internal tandem repeat in patients with de novo acute myeloid leukemia treated with reinforced courses of chemotherapy. Leukemia.

[4] Ravandi F, Kantarjian H, Faderl S, Garcia-Manero G, O'Brien S, Koller C, Pierce S, Brandt M, Kennedy D, Cortes J, Beran M (2010). Outcome of patients with FLT3-mutated acute myeloid leukemia in first relapse. Leuk Res.

[5] Cortes J, Perl AE, Dohner H, Kantarjian H, Martinelli G, Kovacsovics T, Rousselot P, Steffen B, Dombret H, Estey E, Strickland S, Altman JK, Baldus CD, Burnett A, Kramer A, Russell N, Shah NP, Smith CC, Wang ES, Ifrah N, Gammon G, Trone D, Lazzaretto D, Levis M (2018). Quizartinib, an FLT3 inhibitor, as monotherapy in patients with relapsed or refractory acute myeloid leukaemia: an open-label, multicentre, single-arm, phase 2 trial. Lancet Oncol.

[6] Cortes JE, Tallman MS, Schiller GJ, Trone D, Gammon G, Goldberg SL, Perl AE, Marie JP, Martinelli G, Kantarjian HM, Levis MJ (2018). Phase 2b study of 2 dosing regimens of quizartinib monotherapy in *FLT3*-ITD-mutated, relapsed or refractory AML. Blood.

[7] Sanga M, James J, Marini J, Gammon G, Hale C, Li J (2017). An open-label, single-dose, phase 1 study of the absorption, metabolism and excretion of quizartinib, a highly selective and potent FLT3 tyrosine kinase inhibitor, in healthy male subjects, for the treatment of acute myeloid leukemia. Xenobiotica.

[8] Cortes JE, Kantarjian H, Foran JM, Ghirdaladze D, Zodelava M, Borthakur G, Gammon G, Trone D, Armstrong RC, James J, Levis M (2013). Phase I study of quizartinib administered daily to patients with relapsed or refractory acute myeloid leukemia irrespective of FMS-like tyrosine kinase 3-internal tandem duplication status. J Clin Oncol.

[9] Li J, Kankam M, Trone D, Gammon G (2019). Effects of CYP3A inhibitors on the pharmacokinetics of quizartinib, a potent and selective FLT3 inhibitor, and its active metabolite. Br J Clin Pharmacol.

[10] Cortes JE, Khaled S, Martinelli G, Perl AE, Ganguly S, Russell N, Kramer A, Dombret H, Hogge D, Jonas BA, Leung AY, Mehta P, Montesinos P, Radsak M, Sica S, Arunachalam M, Holmes M, Kobayashi K, Namuyinga R, Ge N, Yver A, Zhang Y, Levis MJ (2019). Quizartinib versus salvage chemotherapy in relapsed or refractory FLT3-ITD acute myeloid leukaemia (QuANTUM-R): a multicentre, randomised, controlled, open-label, phase 3 trial. Lancet Oncol.

[11] Vanflyta (quizartinib). Prescribing information. Daiichi Sankyo Co, Ltd; 2019.

[12] Lee M (2009). Basic skills in interpreting laboratory data.

[13] Kang D, Ludwig E, Jaworowicz D, Huang H, Fiedler-Kelly J, Cortes J, Ganguly S, Khaled S, Kramer A, Levis M, Martinelli G, Perl A, Russell N, Abutarif M, Choi Y, Mendell J, Yin O (2020). Population pharmacokinetic analysis of quizartinib in healthy volunteers and patients with relapsed/refractory acute myeloid leukemia. J Clin Pharmacol.

[14] Garnett C, Bonate PL, Dang Q, Ferber G, Huang D, Liu J, Mehrotra D, Riley S, Sager P, Tornoe C, Wang Y (2018). Scientific white paper on concentration–QTc modeling. J Pharmacokinet Pharmacodyn.

[15] Huh Y, Hutmacher MM (2015). Evaluating the use of linear mixed-effect models for inference of the concentration–QTc slope estimate as a surrogate for a biological QTc model. CPT Pharmacometrics Syst Pharmacol.

[16] US Food and Drug Administration (2005) Guidance for industry: E14 clinical evaluation of QT/QTc interval prolongation and proarrhythmic potential for non-antiarrhythmic drugs. https://www.fda.gov/media/71372/download. Accessed 30 Jun 2020

[17] Bonate PL (2013). The effects of active metabolites on parameter estimation in linear mixed effect models of concentration–QT analyses. J Pharmacokinet Pharmacodyn.

[18] Piotrovsky V (2005). Pharmacokinetic–pharmacodynamic modeling in the data analysis and interpretation of drug-induced QT/QTc prolongation. AAPS J.

[19] Weiss JN, Qu Z, Shivkumar K (2017). Electrophysiology of hypokalemia and hyperkalemia. Circ Arrhythm Electrophysiol.

[20] Kang D, Lin KJ, Ludwig E, Yin O (2018) Concentration–QT analysis of quizartinib in patients with relapsed/refractory AML. Presented at: Ninth American Conference on Pharmacometrics; October 7–10, 2018 [Poster M-044]

[21] Meid AD, Bighelli I, Machler S, Mikus G, Carra G, Castellazzi M, Lucii C, Martinotti G, Nose M, Ostuzzi G, Barbui C, Haefeli WE (2017). Combinations of QTc-prolonging drugs: towards disentangling pharmacokinetic and pharmacodynamic effects in their potentially additive nature. Ther Adv Psychopharmacol.

