# Supplementary material for: Concentration–QTc analysis of quizartinib in patients with relapsed/refractory acute myeloid leukemia
Source: Cancer Chemother Pharmacol. 2021 Jan 8;87(4):513–23. doi: 10.1007/s00280-020-04204-y (PMC7946665; doi:10.1007/s00280-020-04204-y)

SUPPLEMENTARY MATERIAL

Concentration-QTc Analysis of Quizartinib in Patients With Relapsed/Refractory Acute Myeloid Leukemia

Dongwoo Kang[1], Elizabeth Ludwig[2], David Jaworowicz[2], Hannah Huang[2], Jill Fiedler-Kelly[2], Jorge Cortes[3], Siddhartha Ganguly[4], Samer Khaled[5], Alwin Krämer[6], Mark Levis[7], Giovanni Martinelli[8], Alexander Perl[9], Nigel Russell[10], Malaz Abutarif[1], Youngsook Choi[1], Ophelia Yin[1]

[1]Daiichi Sankyo, Inc, Basking Ridge, NJ, USA; [2]Cognigen Corporation, a Simulations Plus Company, Buffalo, NY, USA; [3]Georgia Cancer Center at Augusta University, Augusta, GA , USA; [4]University of Kansas Cancer Center, Fairway, KS, USA; [5]City of Hope, Arcadia, CA, USA; [6]Clinical Cooperation Unit Molecular Hematology/Oncology, Department of Internal Medicine V, University of Heidelberg, Heidelberg, Germany; [7]The Sidney Kimmel Comprehensive Cancer Center at Johns Hopkins University, Baltimore, MD, USA; [8]Istituto Scientifico Romagnolo per lo Studio e la Cura dei Tumori (IRST) IRCCS, Meldola, Italy; [9]Division of Hematology and Oncology, Abramson Cancer Center, University of Pennsylvania, Philadelphia, PA, USA; [10]Centre for Clinical Haematology, Nottingham University Hospital, Nottingham, UK

Corresponding author: Dongwoo Kang, dkang@dsi.com

Online Resource 1. Statistical methods.

To investigate the potential impact of demographic and laboratory values on the C-QTc relationship, continuous covariate effects were described using a power model as shown in the following equation: $TVP = \theta_1 \times (X_i / X_{ref})^{\theta 2}$, where X_i is the covariate of interest, X_{ref} is the centering value (eg, median) of that covariate, TVP is the typical value of a model parameter, θ_1 is the typical parameter value for an individual with the median value of the covariate, and θ_2 reflects the change in the typical parameter value as the covariate deviates from the median. For categorical covariates such as sex and race, the change in the typical parameter value was modeled as the fractional change in the typical parameter value as shown in the following equation: $TVP = \theta_1 \times (1 + IND \times \theta_2)$, where θ_1 is the typical parameter value for a reference individual, θ_2 is the fractional change in the typical value for a covariate category, and IND is the indicator variable (IND = 1 if the covariate is present; IND = 0 if the covariate is absent). Covariates were tested one at a time using a stepwise forward addition procedure at a significance of $p<0.01$ (representing a reduction in the minimum objective function value of > 6.63). The most statistically significant covariate was added to the model during each step of forward addition. Remaining covariates were individually added to the new covariate model at each step according to the same procedure and tested for statistical significance using NONMEM. This process was repeated until there were no further covariates that produced significant changes in the objective function value. A stepwise backward elimination process was then applied. Each covariate was removed from each parameter separately to test for statistical significance. A covariate was considered significant if it resulted in an increase in the minimum objective function value of ≥ 10.83 ($\alpha = 0.001$, 1 degree of freedom for χ^2 distribution) when removed from the model. This elimination process was repeated until all nonsignificant covariates were removed.

Online Resource 2. Scatterplot of QTcF versus R-R duration (a) pretreatment and (b) posttreatment. The lines represent smoothing splines fit to the data.

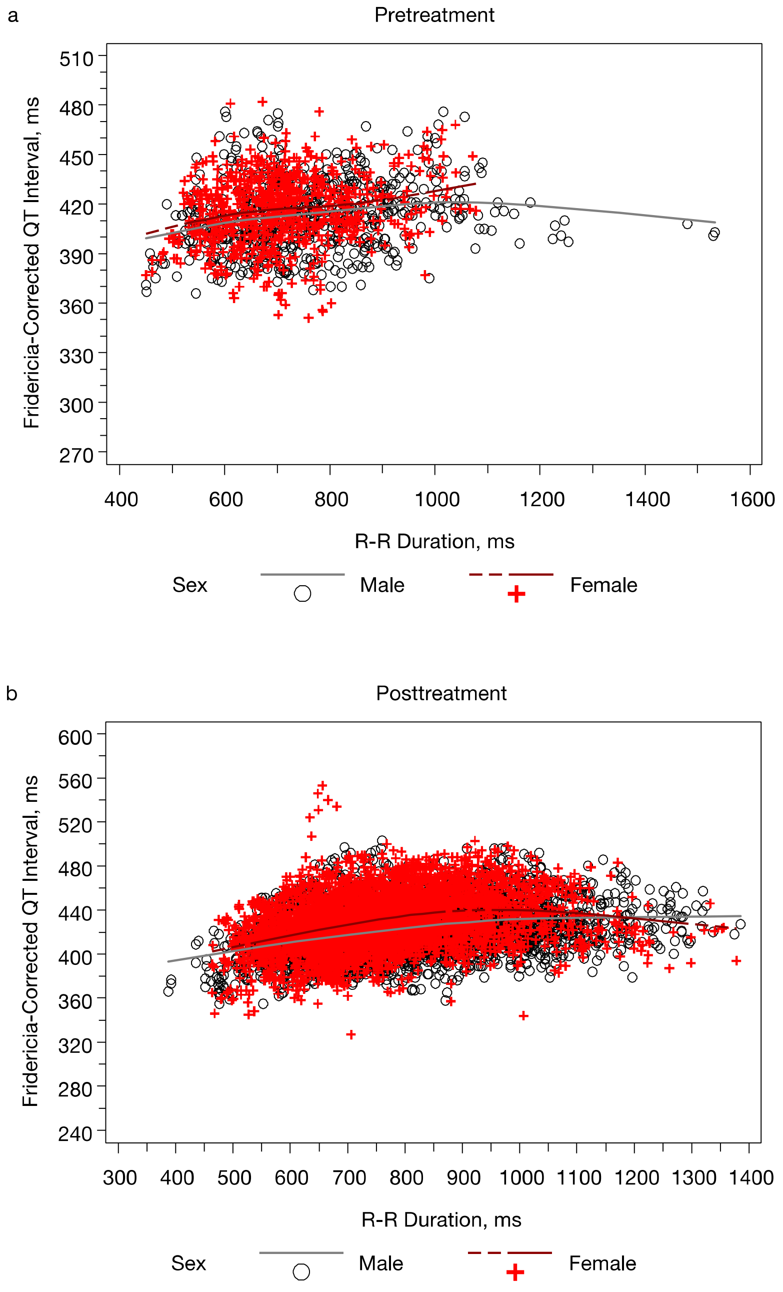

Online Resource 3. Pretreatment QTcF versus sampling clock time within each decile bin of the fixed time effect model. The line represents a smoothing spline fit to the median X and Y values within each X decile bin. The horizontal bars along the X-axis represent the minimum and maximum values for each X decile bin. The vertical bars represent the 90% confidence intervals for the mean of Y values within each X decile bin.

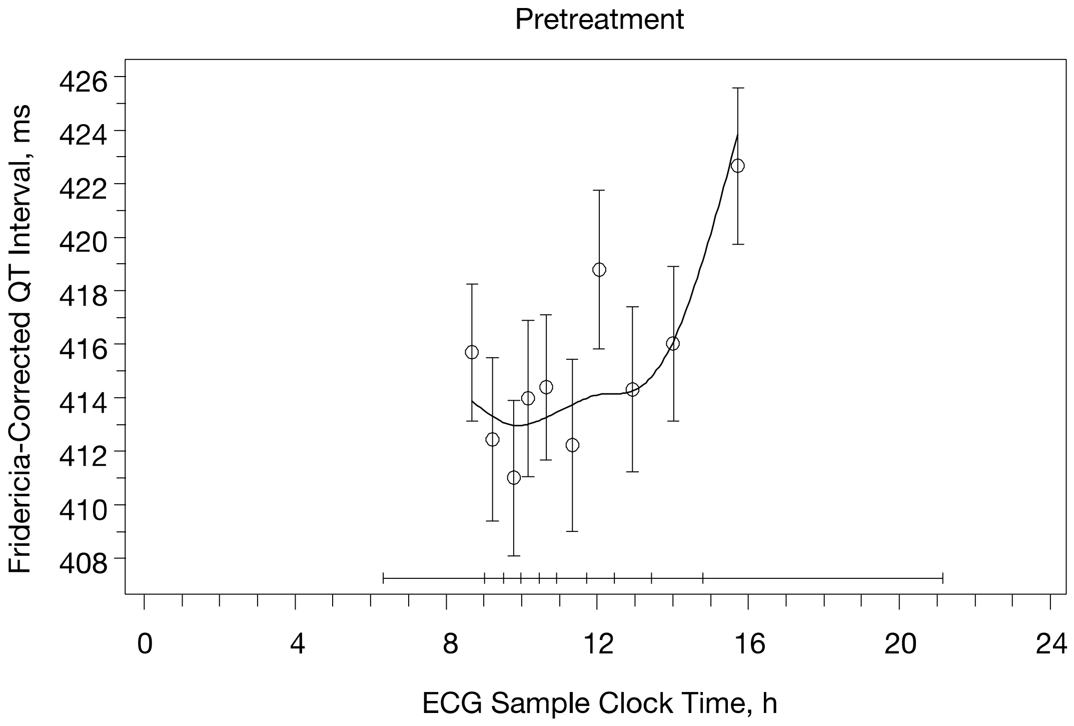

Online Resource 4. Comparison of the objective function value for models with alternative residual variability error and interindividual variability distributions.

Model	Functional Form for Error Model (RV)	Distribution for IIV on Baseline QTcF	Distribution for IIV on E_{max} for Quizartinib	Distribution for IIV on E_{max} for AC886	Objective Function Value
Model A	Additive	Normal	Normal	Normal	17,554.398
Model B	Additive	Normal	Log-normal	Normal	17,559.160
Model C	Additive	Normal	Normal	Log-normal	17,674.776
Model D	Additive	Normal	Log-normal	Log-normal	17,694.763
Model E	Additive	Log-normal	Normal	Normal	17,778.335
Model F	Additive	Log-normal	Log-normal	Normal	Run failed[a]
Model G	Additive	Log-normal	Normal	Log-normal	Run failed[a]
Model H	Additive	Log-normal	Log-normal	Log-normal	17,749.761
Model I (ref)	Proportional	Normal	Normal	Normal	17,534.344
Model J	Proportional	Normal	Log-normal	Normal	17,543.174
Model K	Proportional	Normal	Normal	Log-normal	17,656.339
Model L	Proportional	Normal	Log-normal	Log-normal	17,679.264
Model M	***Proportional***	***Log-normal***	***Normal***	***Normal***	***17,532.178***
Model N	Proportional	Log-normal	Log-normal	Normal	17,540.735
Model O	Proportional	Log-normal	Normal	Log-normal	17,653.942
Model P	Proportional	Log-normal	Log-normal	Log-normal	17,676.357

Model M was the selected model.

AC886, compound code for active metabolite of quizartinib; E_{max}, maximum effect; IIV, interindividual variability; QTcF, QT interval corrected using Fridericia's formula; ref, reference; RV, residual variability.

[a]Run failed due to numerical problems.

Online Resource 5. Histograms of ΔQTcF at the geometric mean $C_{max,ss}$ of quizartinib (401 ng/mL; a) and AC886 (204 ng/mL; b) are shown. Broken red vertical lines are at 10 and 20 ms. At the geometric mean $C_{max,ss}$ of quizartinib, 51.5% and 77.6% of the observations were above 20 and 10 ms, respectively. At the geometric mean $C_{max,ss}$ of AC886, 41.2% and 67.2% of the observations were above 20 and 10 ms, respectively.

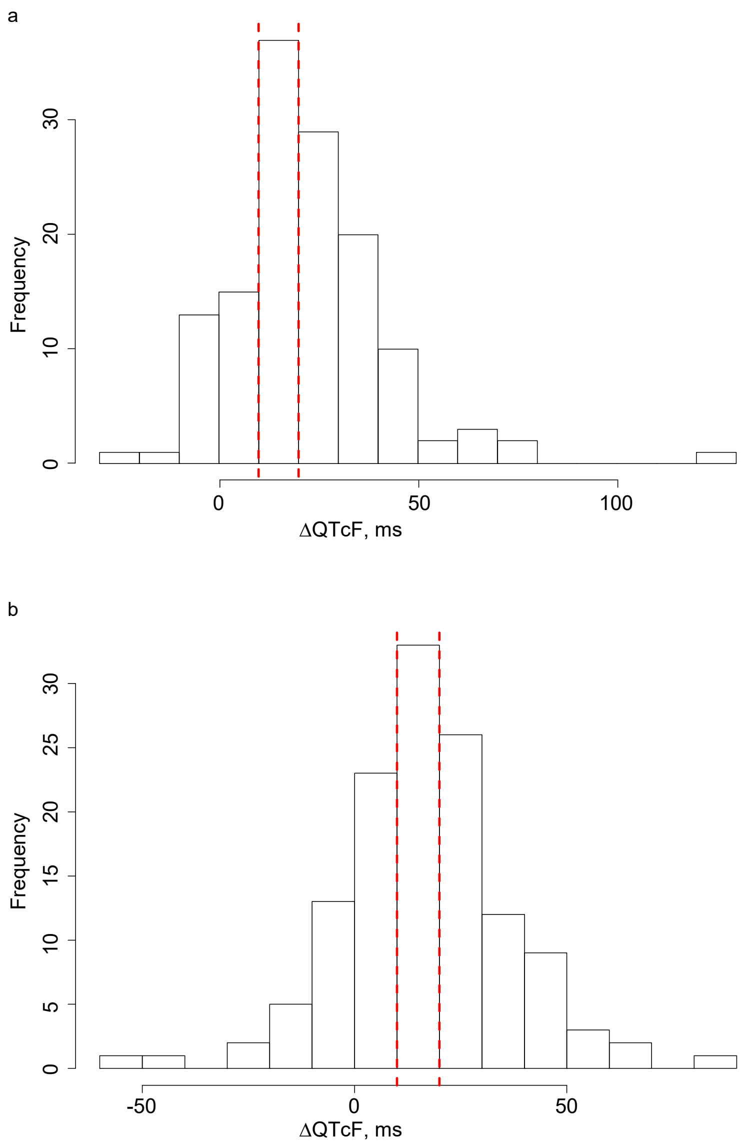

Online Resource 6. List of QT/QTcF-prolonging drugs used in the QuANTUM-R study.

	n	%
**Antibacterials for systemic use**		
Azithromycin	18	7.5
Clarithromycin	3	1.2
Doxycycline	13	5.4
Moxifloxacin	6	2.5
**Antiemetics and antinauseants**		
Granisetron	2	0.8
Ondansetron	66	27.4
Prochlorperazine	25	10.4
**Antifungals for dermatologic use**		
Fluconazole	1	0.4
Ketoconazole	1	0.4
**Antimycotics for systemic use**		
Fluconazole	82	34
Itraconazole	6	2.5
Posaconazole	51	21.2
Voriconazole	28	11.6
Antiprotozoals	19	7.9
Atovaquone	4	1.7
Pentamidine	15	6.2
Immunosuppressants	24	10
Tacrolimus	24	10
**Other dermatologic preparations**		
Tacrolimus	1	0.4
Psychoanaleptics	3	1.2
Amitriptyline	2	0.8
Venlafaxine	1	0.4
Psycholeptics	5	2.1
Chlorpromazine	2	0.8
Haloperidol	4	1.7

Total n = 241.

Online Resource 7. ΔQTcF versus quizartinib concentration in patients who had QT-prolonging drug on and off periods (n = 60).

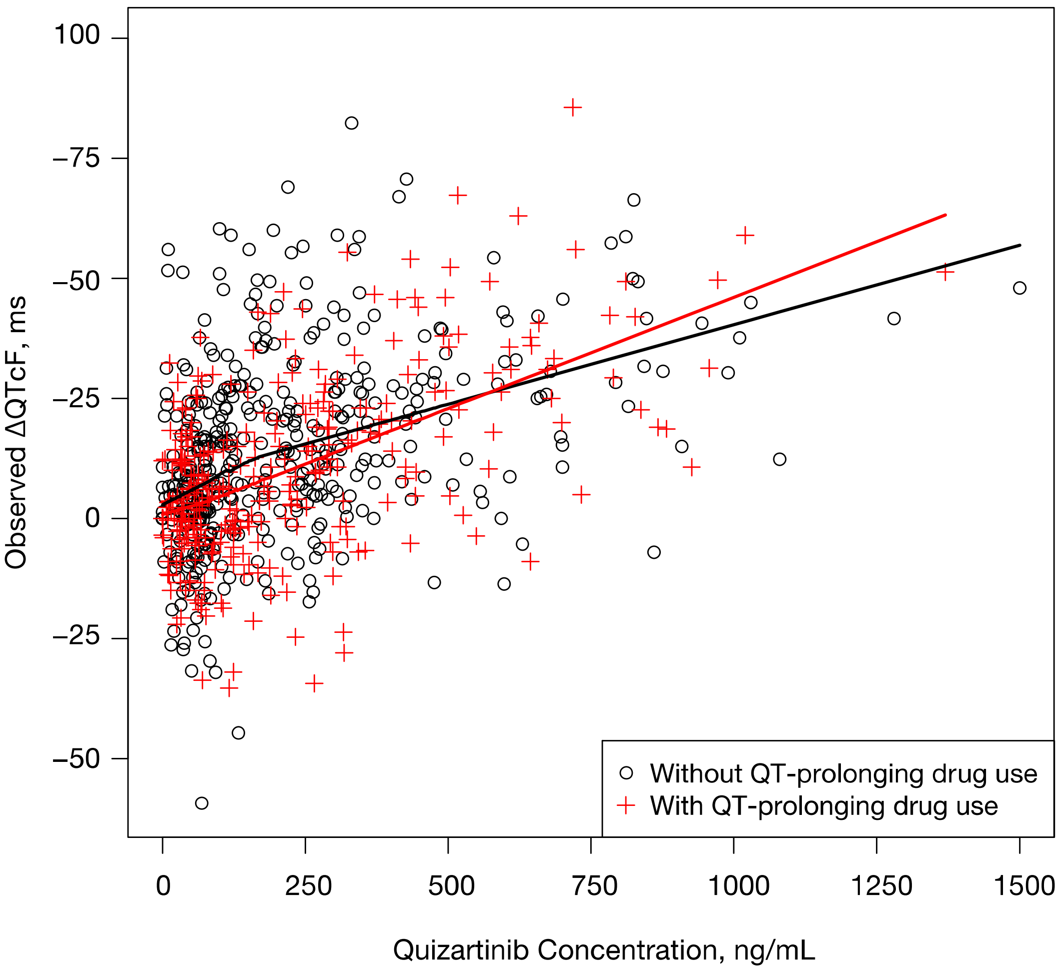

Online Resource 8. Comparison of C-QTc models for (a) quizartinib and (b) AC886. The lines represent the final sigmoid E_{max} model median prediction (and 90% confidence interval in the shaded area), base linear model prediction from the QuANTUM-R study for a typical patient, and final linear model prediction from study 2689-CL-2004 for a typical patient, assuming a metabolite-to-parent ratio of 0.63, 0.63, and 0.5, respectively.

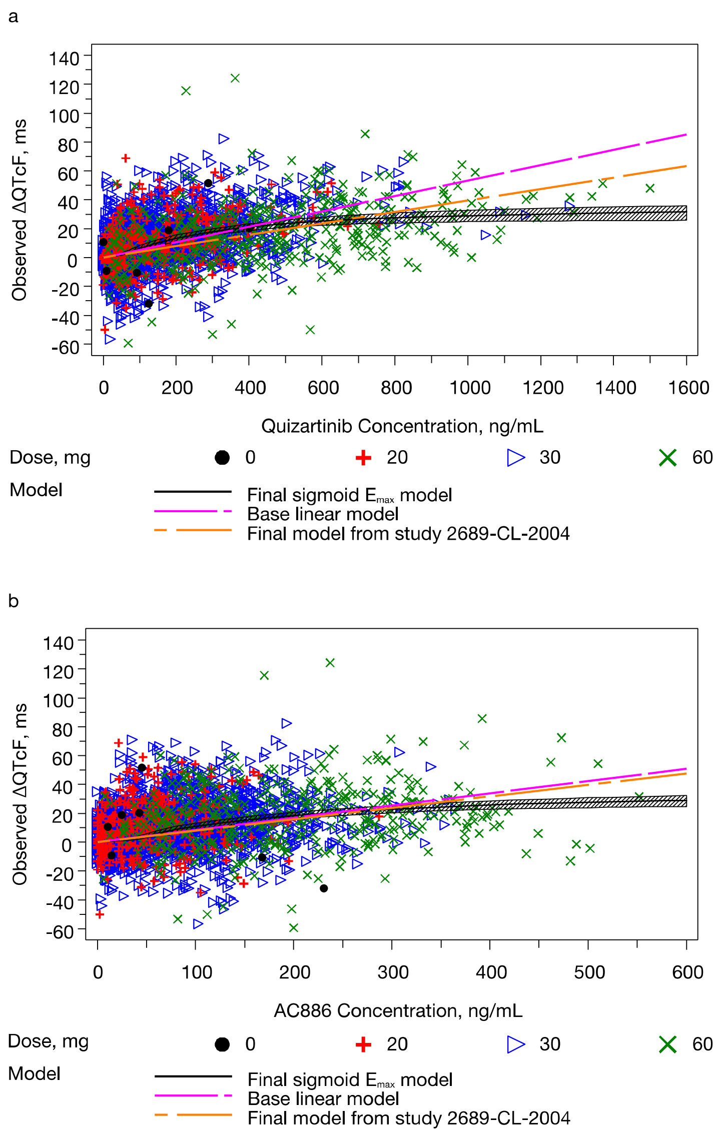

Supplement: Supplementary file 1 — (PDF 559 KB) [file 280_2020_4204_MOESM1_ESM.pdf]
